# Larval exposure to sertraline induces dose- and time-dependent remodeling of neuronal alternative splicing in adult *Drosophila melanogaster*

**DOI:** 10.1007/s11033-026-12452-z

**Published:** 2026-07-30

**Authors:** Luis Felipe Santos-Cruz, Myriam Campos-Aguilar, Laura Castañeda-Partida, María Eugenia Heres-Pulido, Irma Elena Dueñas-García, Elías Piedra-Ibarra, Rafael Jiménez-Flores, Alberto Ponciano-Gomez

**Affiliations:** 1 Genetics Toxicology, Biology, Facultad de Estudios Superiores Iztacala, Universidad Nacional Autónoma de México, Los Barrios No. 1, Los Reyes Iztacala, Tlalnepantla, 54090 Mexico; 2 Immunology Laboratory (UMF), Facultad de Estudios Superiores Iztacala, Universidad Nacional Autónoma de México, Los Barrios No. 1, Los Reyes Iztacala, Tlalnepantla, 54090 Mexico; 3 Plant Physiology (UBIPRO), Facultad de Estudios Superiores Iztacala, Universidad Nacional Autónoma de México, Los Barrios No. 1, Los Reyes Iztacala, Tlalnepantla, 54090 Mexico

**Keywords:** Alternative splicing, Sertraline, Neuroplasticity, *Drosophila melanogaster*, RNA-seq

## Abstract

**Background:**

Alternative splicing is a central procedure that increases the variety of the transcriptome and helps regulate several neuronal processes. Various pharmacological factors have the capacity to alter splicing patterns, which could potentially affect cellular function. Sertraline, a selective serotonin reuptake inhibitor widely used in the treatment of neuropsychiatric disorders, also regulates intracellular pathways linked with calcium signaling and other cellular processes. However, information about its potential impact on the post-transcriptional regulation of the transcriptome is still insufficient.

**Methods and Results:**

In this study, we analyzed whether exposure to sertraline changes alternative splicing patterns in the neural transcriptome of *Drosophila melanogaster.* Third-instar larvae were exposed to two concentrations of the drug during different periods of time, and the RNA obtained from adult heads was analyzed by RNA sequencing (RNA-seq). The evaluation of differential splicing revealed modifications that depend on the experimental condition in exon usage, including exon skipping, intron retention, and alternative splice-site selection. The affected genes showed functional enrichment in processes related to ion transport, synaptic organization, and neuronal signaling. The in silico reconstruction and translation of representative isoforms indicated possible modifications in protein architecture, including predicted loss of domains or truncations.

**Conclusions:**

Taken together, these results indicate that sertraline can remodel alternative splicing in the neuronal transcriptome in a manner dependent on dose and exposure time. These findings suggest a possible additional mechanism through which larval sertraline exposure could influence neuronal function via persistent remodeling of RNA processing in adult neural tissue, as inferred from RNA-seq-based transcriptomic analyses.

**Supplementary Information:**

The online version contains supplementary material available at 10.1007/s11033-026-12452-z.

## Introduction

Sertraline is one of the most widely used selective serotonin reuptake inhibitors (SSRIs) for the treatment of mood disorders. SSRIs exert their primary pharmacological action by inhibiting the serotonin transporter (SERT), thereby increasing extracellular serotonin availability and enhancing serotonergic neurotransmission [1]. However, its action is not limited to the serotonergic system: it also modulates Ca²⁺ homeostasis, interacts with σ1 receptors, and influences the synthesis of neurosteroids, directly affecting neuronal excitability and intracellular signaling dynamics [1, 2]. Together, these effects position sertraline as a broader modulator of neuronal functional state, extending beyond monoaminergic neurotransmission.

The stability and adaptation of neural circuits depend on the ability of neurons to adjust the efficiency of synaptic transmission and the organization of their subcellular domains. It has been documented that sertraline can remodel synaptic structural elements and modify the balance between stability and flexibility, influencing mechanisms associated with medium- and long-term plasticity [3]. However, the molecular processes underlying these structural and functional adjustments remain insufficiently characterized.

Among the mechanisms that allow the fine adjustment of neuronal function, alternative splicing stands out for its capacity to generate protein isoforms with distinct regulatory and biophysical properties. In the nervous system, this mechanism is essential for the modulation of ion channels, signaling proteins, and components of the synaptic matrix, allowing the sensitivity and dynamics of neural circuits to be adjusted according to physiological context [4]. In *Drosophila melanogaster*, pharmacological exposure has been shown to induce persistent splicing changes that persist into adulthood, affecting synaptic organization and neuronal transmission [5]. However, the influence of dose and exposure duration on the resulting isoform repertoires and their potential structural consequences remains poorly understood.

Given that sertraline acts on pathways related to Ca²⁺ regulation, neuronal excitability, and synaptic organization, it is plausible that it also modulates the splicing mechanisms governing these processes. Therefore, this study evaluated how the dose and duration of sertraline exposure modify the isoform repertoire in the neural transcriptome of *Drosophila melanogaster*, with particular emphasis on the predicted structural consequences associated with the observed transcriptomic changes.

## Materials and methods

### Experimental design and treatments

Third-instar larvae (72 ± 4 h old) from the wild-type Canton-S strain of *Drosophila melanogaster* were exposed to sertraline (Pfizer, Altruline^®^, New York, USA) dissolved in 1% DMSO, sertraline doses of 50 mg or 200 mg were incorporated into culture vials containing 33 mL of larval food. Negative control groups received MilliQ water, whereas vehicle-control groups received 1% DMSO under identical experimental conditions. Four experimental conditions were established to evaluate the effects of dose and exposure time: treatments with 50 mg for 24 and 48 h and treatments with 200 mg for 24 and 48 h. Each experimental group consisted of 100 individuals maintained under controlled environmental conditions (25 °C, 65% relative humidity, and a 12:12 h light: dark photoperiod). Approximately 30 min after eclosion, newly emerged adult flies were allowed an additional four hours before collection. Flies were then anesthetized by cold exposure and immediately preserved on dry ice until further processing.

### Neural tissue isolation

For transcriptomic analyses, adult fly heads from 100 individuals per experimental group were collected and processed. Two independent biological replicates were generated for each experimental condition, each consisting of a separate pool of 100 adult fly heads. Only adult males were used; sex was determined based on external morphological characteristics shortly after eclosion. Heads were dissected in cold phosphate-buffered saline (PBS, pH 7.0) under sterile conditions using pre-chilled instruments to minimize RNA degradation. Samples were immediately transferred to pre-cooled microtubes and stored at − 70 °C until further processing. The entire procedure, including sacrifice, dissection, and storage, was completed within a maximum of 30 min to preserve RNA integrity and minimize potential alterations in gene expression.

### RNA extraction and quality control

Total RNA extraction was performed using the RNeasy Kit (QIAGEN, Venlo, The Netherlands) according to the manufacturer’s protocol. Messenger RNA was subsequently purified using the RNeasy Pure mRNA Bead Kit (QIAGEN, Venlo, The Netherlands). RNA concentration and purity were assessed using a NanoDrop 2000 spectrophotometer, ensuring a minimum concentration of 5 ng/µL, a volume not lower than 20 µL, and an OD260/280 absorbance ratio of 2.0. All procedures were performed in duplicate for each sample to ensure reproducibility and consistency.

### RNA-Seq sequencing

Transcriptomic profiling was performed by RNA sequencing (RNA-Seq) on the Illumina NovaSeq 6000 platform, with services provided by Novogene Corporation Inc. (Sacramento, CA, USA). RNA samples obtained from both control groups (MilliQ water and 1% DMSO vehicle) and sertraline-exposed groups were processed using the same sequencing workflow. Library preparation, quality control, and sequencing parameters followed the standardized protocols implemented by the company. Raw sequencing data were delivered in FASTQ format for subsequent bioinformatic analyses.

### Bioinformatic analysis

Following sequencing, raw data in FASTQ format were processed using fastp to remove adapters, filter out low-quality reads, and calculate Q20, Q30, and GC content. All subsequent analyses were performed using these quality-filtered sequences and their corresponding metrics.

Read alignment was performed with HISAT2 v2.0.5 using the *Drosophila melanogaster* reference genome (FlyBase r6.32) and gene annotation files downloaded directly from the official FlyBase repository. A reference genome index was built with HISAT2, and paired-end clean reads were aligned using the same software. This aligner was selected because it incorporates splice junction information from gene annotations, providing higher mapping accuracy and improved detection of splicing sites compared with non–splice-aware tools.

Aligned regions were classified as exonic, intronic, or intergenic. Exon-mapped reads represented the most abundant type, as expected in well-annotated genomes, whereas intron-mapped reads could result from pre-mRNA contamination or from intron-retention events related to alternative splicing.

Baseline transcript expression levels were quantified with StringTie2, which calculated RPKM values for each identified gene or transcript. Alternative splicing events were quantified from junction reads, which are essential for determining exon connectivity within a transcript, and were identified and counted using CIGAR string information from the BAM alignment files.

Comprehensive alternative splicing analysis was performed using rMATS v3.2.5, which classified the major event types, including skipped exon (SE), mutually exclusive exon (MXE), alternative 5′ splice site (A5SS), alternative 3′ splice site (A3SS), and retained intron (RI). The proportion of exon inclusion (IncLevel) was calculated as an indicator of splicing frequency for each event.

Statistical analysis of differential splicing events was performed using a model based on the edgeR v4.0 package, considering events with a false discovery rate (FDR) below 0.05 as significant. This threshold reduced the likelihood of false positives and increased robustness in detecting differences in isoform usage and splicing frequency.

Pairwise similarity between sets of alternatively spliced genes was evaluated using the Jaccard index, calculated as the number of shared genes divided by the total number of unique genes present across both comparisons.

### Reconstruction of mRNA isoforms

Reconstruction of mRNA isoforms resulting from alternative splicing events was performed using the GTF files generated by rMATS, based on the *Drosophila melanogaster* reference genome and annotation (FlyBase release 6.32). Each transcript was mapped according to the genomic coordinates of its exons, and full-length and truncated isoforms were assembled independently as computational models derived from RNA-seq data. Total mRNA length and exon order were computed using custom scripts written in Python v3.11, employing the pandas, numpy, and biopython libraries for structural data processing and sequence management.

Graphical representations of mRNA architectures were generated using the matplotlib package, with scales proportional to nucleotide length and sequential exon numbering.

### Isoform translation and evaluation of protein domains

The previously reconstructed mRNA isoforms were translated in silico to obtain their corresponding protein sequences, using the annotated reading frame from *Drosophila melanogaster* (FlyBase r6.32). These translated sequences were used for predictive structural analyses of putative protein products. Translation was performed in Python 3.11 using the Bio.Seq module from Biopython (v1.83) with the standard genetic code. For each isoform, the nucleotide sequence was read from the start codon to the first in-frame stop codon, adjusting cases in which alternative splicing introduced premature stops or frame shifts.

Protein domain coordinates were retrieved from UniProtKB and FlyBase, which provide structural and functional annotations for *Drosophila* proteins. Each domain was assessed according to its overlap with the translated protein sequence and classified as “retained” when the full domain interval (start–end in aa) remained intact within the isoform, “truncated” when partial overlap with terminal loss was observed, and “lost” when the corresponding region was absent.

Based on this classification, predicted protein length and domain status were determined for each isoform, and linear maps were generated to depict conserved, truncated, or missing regions. All processing was implemented in Python 3.11 using pandas (v2.2.2) and numpy (v1.26) for tabular and coordinate handling, biopython (v1.83) for translation and sequence management, and matplotlib (v3.9) for graphical rendering.

## Results

### Alternative splicing events induced by sertraline exposure

**Fig. 1:**
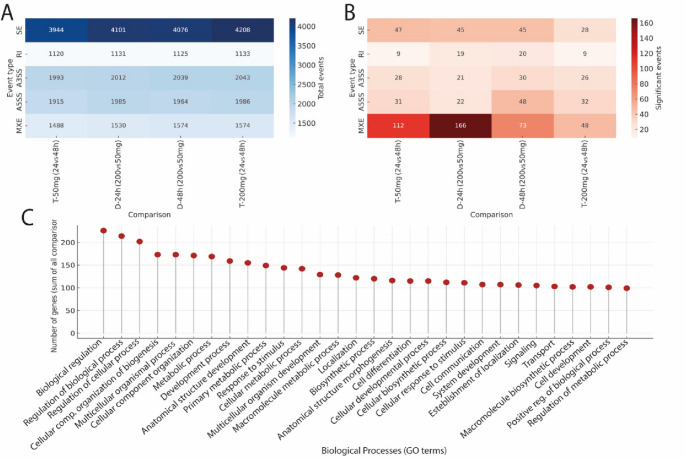
Alternative splicing events induced by sertraline exposure. (**A**) Distribution of the five types of alternative splicing events detected with rMATS: skipping exon (SE), mutually exclusive exons (MXE), alternative 5’ splice sites (A5SS), alternative 3’ splice sites (A3SS), and retention intron (RI), shown for each experimental comparison. (**B**) Heatmaps displaying the inclusion levels (IncLevel) of selected genes with significant splicing differences, ordered by experimental condition and grouped by event type. Color scales indicate the relative exon inclusion across samples. (**C**) Biological processes (Gene Ontology, Biological Process category) associated with alternatively spliced genes across all comparisons, represented by the 30 most frequent terms

Across the four experimental comparisons, rMATS detected a total of 42,610 alternative splicing events (Fig. 1A). After applying the significance criteria, 850 events remained significantly modulated by sertraline exposure. Among these significant events, mutually exclusive exons (MXE) were the most frequent event type (390 events), followed by skipped exons (SE; 165 events), alternative 5′ splice sites (A5SS; 133 events), alternative 3′ splice sites (A3SS; 105 events), and retained introns (RI; 57 events). Among the experimental comparisons, the highest number of significant events was observed in the D-24 h (200vs50 mg) comparison (268 events), whereas the T200mg (24vs48h) comparison exhibited the lowest number of alterations (142 events).

Once significant alternative splicing events were identified, genes displaying differential splicing were evaluated based on IncLevel values. In this analysis, mutually exclusive exons (MXE) again represented the predominant event type, with IncLevel values clearly distinguishing the experimental conditions. The comparison showing the largest number of differentially spliced genes was D-24 h (200vs50 mg), which displayed 166 MXE events, 45 SE, 22 A5SS, 21 A3SS, and 19 RI, whereas T200mg (24vs48h) comparison exhibited the lowest number of alterations (48 MXE, 28 SE, 32 A5SS, 26 A3SS, and 9 RI) (Fig. 1B).

Functional annotation of the affected genes revealed that the most represented Gene Ontology terms belonged to the Biological Process category. The most frequent processes were biological regulation (226 genes), regulation of biological process (214), and regulation of cellular process (202), followed by terms associated with cellular component organization and developmental processes (Fig. 1C).

**Fig. 2:**
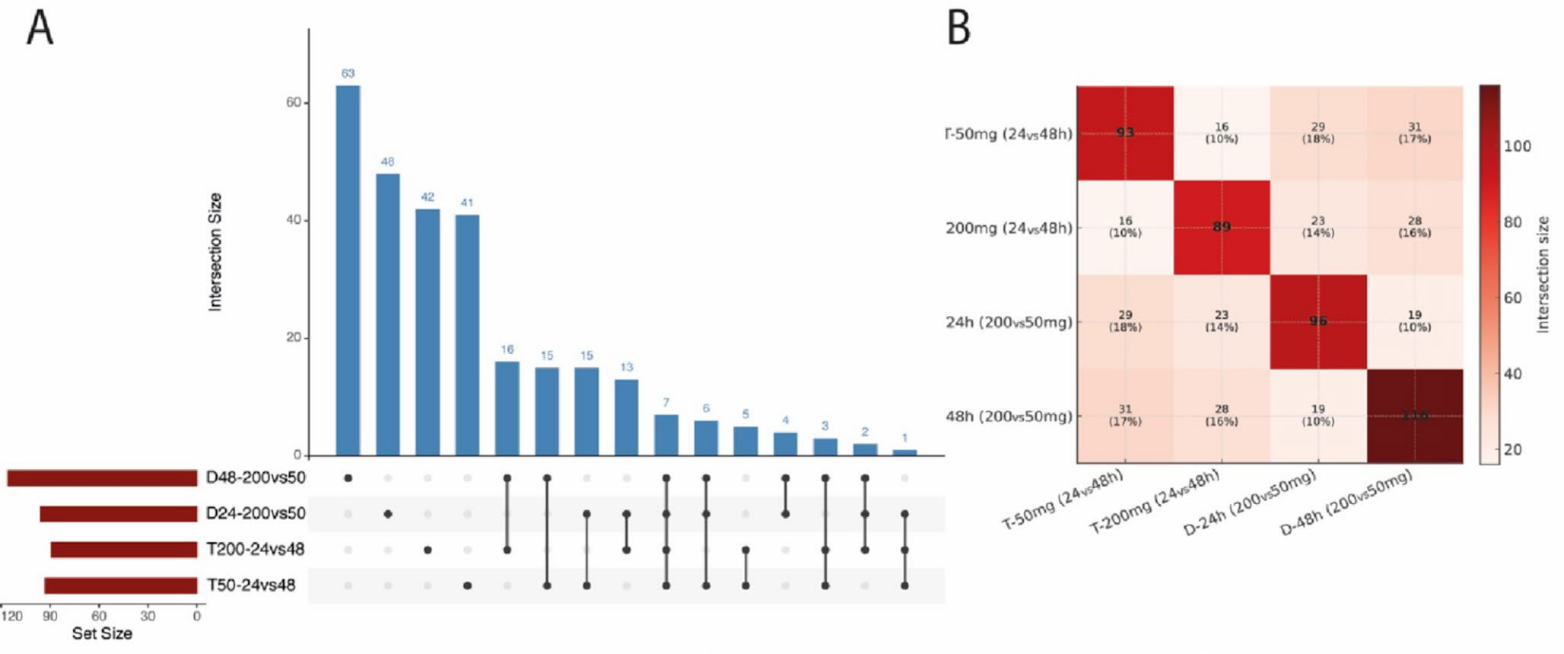
Overlap and similarity of alternatively spliced genes across comparisons. (**A**) UpSet plot displaying the intersections of genes with significant splicing changes (FDR ≤ 0.05) among the four experimental comparisons. Horizontal bars on the left indicate the total number of significant genes per comparison, while vertical bars on top represent the size of each intersection. Filled dots below the bars denote which comparisons are included in each intersection. (**B**) Heatmap summarizing pairwise overlaps between comparisons. Diagonal cells show the total number of significant genes per comparison, while off-diagonal cells indicate the number of genes shared between comparisons, with Jaccard similarity percentages shown in parentheses. Color intensity reflects the size of the intersection

## Overlap and similarity of alternatively spliced genes across comparisons

Intersection analysis revealed that the sets of genes with significant alternative splicing (FDR ≤ 0.05) displayed distinct patterns in each comparison (Fig. 2A). The largest exclusive group was observed in D-48 h (200vs50 mg), with 63 genes detected only under this condition, followed by D-24 h (200vs50 mg) with 48, T200mg (24vs48h) with 42, and T50mg (24vs48h) with 41. In addition to these exclusive sets, specific intersections were identified, including 16 genes shared between D-48 h and T200mg (24vs48h), and 15 genes shared between D-24 h and T50mg (24vs48h). Notably, seven genes were common to all four comparisons.

Pairwise overlap analysis confirmed these relationships (Fig. 2B). According to the Jaccard index, the highest relative similarity was observed between T50mg (24vs48h) and D-24 h (200vs50 mg), which shared 29 genes (Jaccard index, 18%). In contrast, the lowest similarity values were observed for T50mg (24vs48h) versus T-200 mg (24vs48h) (16 genes; Jaccard index, 10%) and for D-24 h versus D-48 h (200vs50mg) (19 genes; Jaccard index, 10%). Overall, these results indicate that although each condition generated largely distinct sets of alternatively spliced genes, partial overlaps reflect conserved responses to sertraline exposure.

## Exclusive splicing events across experimental comparisons

**Fig. 3:**
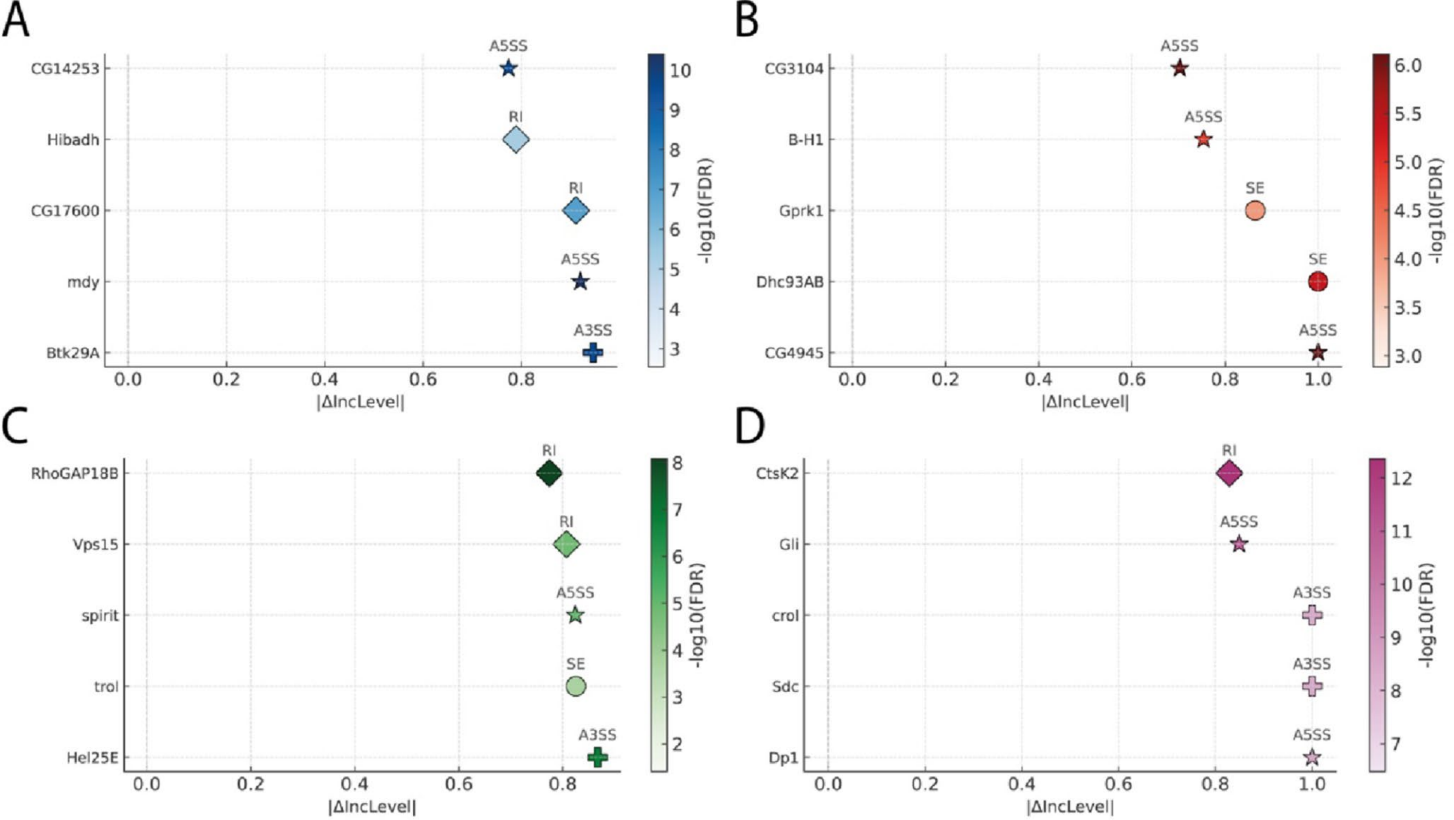
Exclusive alternatively spliced genes in each experimental comparison. Each panel shows the five most significant genes (FDR ≤ 0.05) identified exclusively in each comparison: (**A**) T50mg (24vs48h; blue), (**B**) T200mg (24vs48h; red), (**C**) D-24 h (200vs50 mg; green), (**D**) D-48 h (200vs50 mg; purple). The X-axis represents the magnitude of exon inclusion changes (|ΔIncLevel|), while the Y-axis indicates the gene name. Color encodes statistical significance (− log10 FDR), and marker shape denotes the type of splicing event: skipping exon (SE), mutually exclusive exons (MXE), retention intron (RI), alternative 5′ splice sites (A5SS), and alternative 3′ splice sites (A3SS)

The previous intersection analysis revealed the presence of exclusive sets of genes with alternative splicing events under each condition. Based on the sashimi plots of each gene (Supplementary Figs. 1–4), comparative panels were constructed to summarize the five most significant events (FDR ≤ 0.05) in each case (Fig. 3).

In T50mg (24vs48h), exclusive changes were detected in Btk29A (A3SS, |ΔIncLevel|=0.946, FDR = 5.41 × 10⁻¹⁰), mdy (A5SS, 0.920, 3.99 × 10⁻¹¹), CG17600 (RI, 0.911, 8.08 × 10⁻⁶), Hibadh (RI, 0.789, 2.83 × 10⁻³), and CG14253 (A5SS, 0.774, 8.27 × 10⁻¹⁰) (Fig. 3A; Supplementary Fig. 1). For T200mg (24vs48h), the exclusive set included CG4945 (A5SS, |ΔIncLevel|=1.000, FDR = 7.73 × 10⁻⁷), Dhc93AB (SE, 1.000, 1.15 × 10⁻⁵), Gprk1 (SE, 0.865, 1.30 × 10⁻³), B-H1 (A5SS, 0.754, 6.59 × 10⁻⁵), and CG3104 (A5SS, 0.703, 7.73 × 10⁻⁷) (Fig. 3B; Supplementary Fig. 2).

Similarly, in D-24 h (200vs50 mg) the exclusive genes were Hel25E (A3SS, |ΔIncLevel|=0.867, FDR = 7.93 × 10⁻⁷), trol (SE, 0.825, 3.88 × 10⁻²), spirit (A5SS, 0.824, 3.95 × 10⁻⁴), Vps15 (RI, 0.807, 7.62 × 10⁻⁴), and RhoGAP18B (RI, 0.774, 8.29 × 10⁻⁹) (Fig. 3C; Supplementary Fig. 3). Finally, in D-48 h (200vs50 mg) the exclusive set comprised Dp1 (A5SS, |ΔIncLevel|=1.000, FDR = 1.39 × 10⁻⁷), Sdc (A3SS, 1.000, 3.33 × 10⁻⁷), crol (A3SS, 1.000, 3.33 × 10⁻⁷), Gli (A5SS, 0.849, 7.78 × 10⁻¹¹), and CtsK2 (RI, 0.829, 4.37 × 10⁻¹³) (Fig. 3D; Supplementary Fig. 4).

Overall, these exclusive repertoires were characterized by a predominance of alternative splice site events (A3SS and A5SS), followed by retention intron (RI) and skipping exon (SE), while mutually exclusive exons (MXE) were not observed.

## Changes in mRNA architecture induced by sertraline

**Fig. 4:**
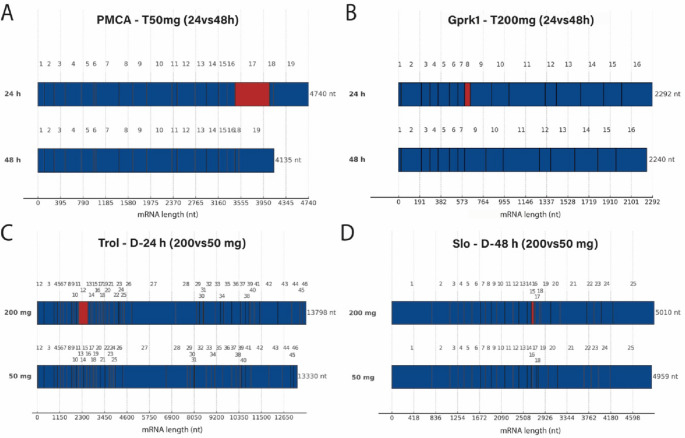
Changes in mRNA architecture induced by sertraline. In (**a**), the isoforms of PMCA are shown comparing T50mg (24and48h); in (**b**), those of Gprk1 under T200mg (24and48h); in (**c**), those of Trol after the dose reduction D-24 h (200vs50 mg); and in (**d**), those of Slo at D-48 h (200vs50 mg). Each bar represents a complete transcript, with exons numbered in blue and differential splicing events highlighted in red. The labels on the left indicate the corresponding dose, and the values at the right end show the total mRNA length in nucleotides

Once the genes with alternative splicing events were identified in each comparison, genes displaying clear modifications in mRNA architecture were selected for detailed analysis. Selection was based on the presence of evident structural alterations in the reconstructed transcripts and their predicted effects on protein architecture, rather than on the magnitude of the statistical significance alone. The results revealed representative predicted alterations in transcript architecture in four genes (PMCA, Gprk1, Trol, and Slo), each corresponding to a distinct experimental comparison (Fig. 4A–D).

In the PMCA gene, corresponding to the comparison T50mg (24 hvs48h), a skipping exon event involving the loss of exon 17 was identified (Fig. 4A). The form observed at 24 h retains the 19 consecutive exons (E1–E19), whereas the form at 48 h contains 18 exons after the complete omission of exon 17. In the 24 h transcript, this exon is located between positions 3454 and 4058 nt, with a length of 605 nt. The total transcript at 24 h reaches 4740 nt, while that at 48 h comprises 4135 nt (Fig. 4A).

In the Gprk1 gene, corresponding to the comparison T200mg (24vs48h), a skipping exon event involving the loss of exon 8 was identified (Fig. 4B). The form observed at 24 h contains 16 exons (E1–E16), whereas the form at 48 h retains 15 exons after the complete omission of exon 8. In the 24 h transcript, this exon is located between positions 597 and 648 nt, with a length of 52 nt. The total transcript at 24 h reaches 2292 nt, while that at 48 h comprises 2240 nt (Fig. 4B).

In the Trol gene, corresponding to the comparison D–24 h (200vs50 mg), a skipping exon event involving the loss of exon 12 was identified (Fig. 4C). The form observed at 200 mg contains 46 exons (E1–E46), whereas the form corresponding to 50 mg retains 45 exons after the complete omission of this exon. In the 200 mg transcript, exon 12 is located between positions 2136 and 2603 nt, with a length of 468 nt. The total transcript at 200 mg reaches 13 798 nt, while that at 50 mg comprises 13 330 nt (Fig. 4C).

In the Slo gene, corresponding to the comparison D–48 h (200vs50 mg), a skipping exon event involving the loss of exon 15 was identified (Fig. 4D). The form observed at 200 mg contains 25 exons and reaches 5010 nt, whereas the form corresponding to 50 mg retains 24 exons and comprises 4959 nt. The skipped exon is 51 nt long (Fig. 4D).

### Predicted changes in protein architecture associated with alternative splicing

**Fig. 5:**
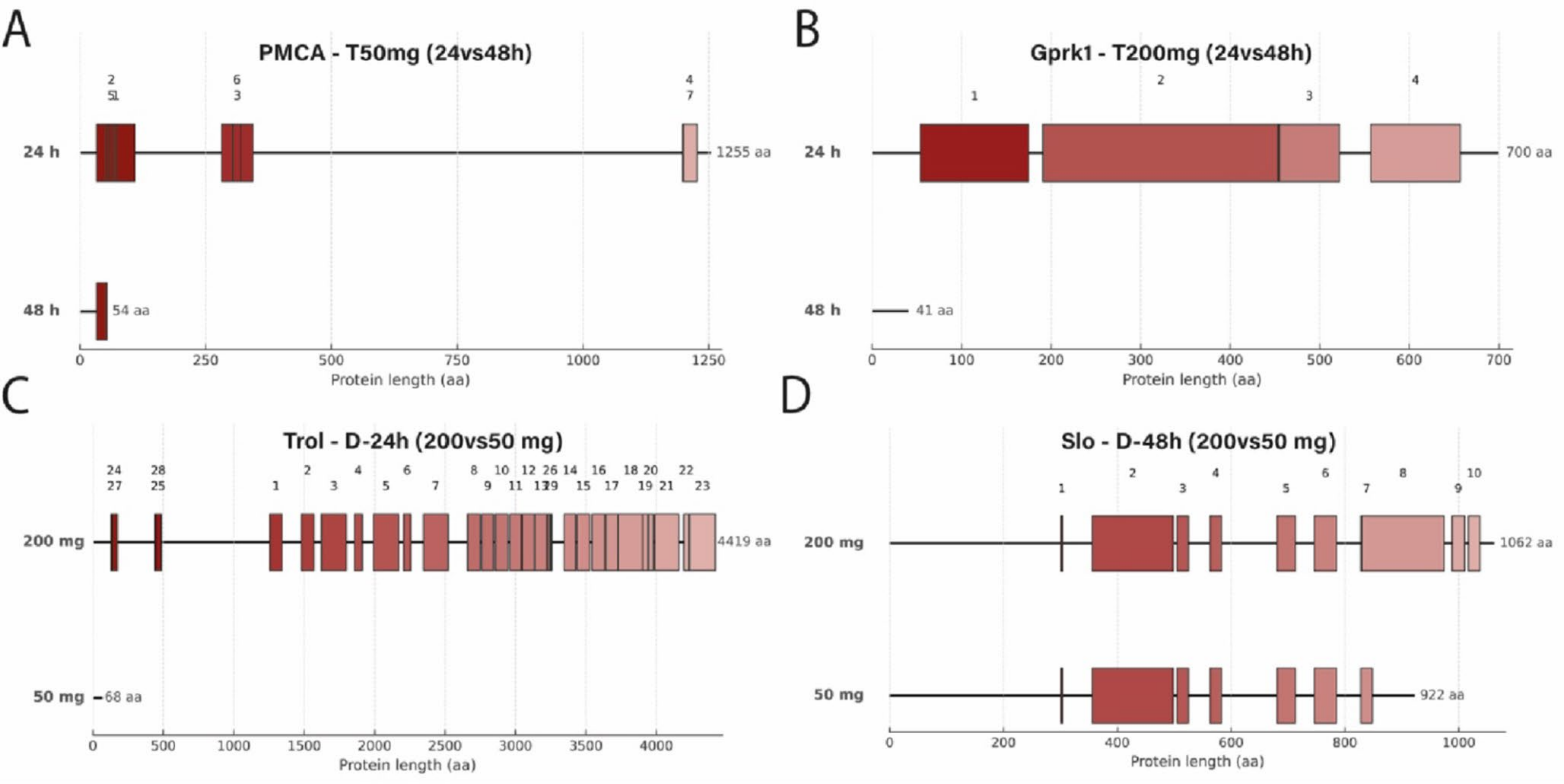
Predicted changes in protein architecture associated with sertraline-induced alternative splicing. In (**a**), the PMCA isoforms are shown comparing T50mg (24vs48h); in (**b**), those of Gprk1 under T200mg (24vs48h); in (**c**), those of Trol after the dose reduction D-24 h (200vs50 mg); and in (**d**), those of Slo at D-48 h (200vs50 mg). Each bar represents a complete protein isoform, with functional domains indicated in shades of red according to their relative position along the amino acid sequence. The labels on the left indicate the corresponding dose, and the values at the right end show the total protein length in amino acids

Based on the alternative splicing events identified in the representative transcripts, predicted structural consequences at the protein level were evaluated *in silico* (Fig. 5). Comparative analysis of the isoforms suggested differences in protein length and domain composition, including the loss or truncation of annotated domains in some reconstructed isoforms.

In the PMCA gene, corresponding to the comparison T50mg (24vs48h), the splicing change was predicted to alter the organization of the encoded protein (Fig. 5A). The form observed at 24 h retains the N-terminal Cation-transporting P-type ATPase domain (aa 33–109), together with three disordered regions (aa 53–72, 282–344, and 1198–1227) and three compositionally biased segments enriched in polar, basic, and acidic residues (aa 58–67, 304–320, and 1199–1227). In contrast, the 48 h form preserves only two truncated segments—the N-terminal domain and one disordered region (aa 33–109 and 53–72)—and was predicted to lose the remaining five functional regions, suggesting a marked reduction in the protein’s structural complexity (Fig. 5A).

In contrast to PMCA, the splicing change in Gprk1 under the T200mg (24vs48h) condition was predicted to result in a complete loss of the protein’s functional domains (Fig. 5B). The 24 h form exhibits four well-defined domains: the RGS (aa 54–175), the Protein kinase (aa 191–454), the AGC-kinase C-terminal (aa 455–522), and the PH (aa 557–657). In contrast, the 48 h form was predicted to lack all of these domains, indicating the absence of the annotated catalytic and regulatory regions identified in the 24 h isoform (Fig. 5B).

The alternative splicing event identified in Trol during the D-24 h (200vs50 mg) comparison was predicted to result in the complete loss of functional protein domains, with the total protein length reduced to 68 aa (Fig. 5C). The 200 mg isoform retains 29 structural regions, including multiple Ig-like domains, Laminin IV type A and Laminin G domains, and EGF-like motifs, as well as disordered regions and compositionally biased segments. In contrast, the 50 mg isoform was predicted to lack all these elements, suggesting the absence of several annotated domains present in the longer isoform (Fig. 5C).

In Slo, the alternative splicing event induced after the dose reduction D–48 h (200vs50 mg) was predicted to generate a shorter protein variant that retains the main structural domains but loses key regions of the C-terminal end (Fig. 5D). The full-length isoform (200 mg) maintains the RCK N-terminal 1 (356–498 aa) and RCK N-terminal 2 (830–974 aa) domains, together with segments S7 (505–525), S8 (563–583), and S9 (830–848), as well as two disordered regions located between 681–713 and 746–785 aa.

In contrast, the shortened form corresponding to the 50 mg treatment retains the RCK domains and segments S7–S9 but displays a truncated RCK N-terminal 2 (830–922 aa) and completely loses the “Calcium bowl” (988–1010 aa), segment S10 (1017–1037 aa), and the disordered region 1170–1200 aa. These changes suggest alterations in the architecture of the calcium-sensitive regulatory tail, although their functional consequences remain to be determined (Fig. 5D).

## Discussion

Selective serotonin reuptake inhibitors exert effects that extend beyond acute increases in neurotransmission, involving slow plastic and transcriptional adaptations in the central nervous system [6]. In *Drosophila*, neuronal alternative splicing generates extensive isoform diversity, particularly in ion channels and synaptic proteins, thereby tuning neuronal excitability and circuit function [7]. Given previous evidence that sertraline reconfigures neuronal splicing in *Drosophila* [5], our findings further demonstrate that sertraline induces a dose- and time-dependent remodeling of alternative splicing that persists from larval exposure into adulthood.

Our results demonstrate a broad dose- and time-dependent remodeling of alternative splicing. The affected genes were enriched in biological regulation, cellular organization, and developmental processes, indicating that these changes reflect coordinated transcriptomic responses rather than random splicing variation. Importantly, the temporal separation between larval exposure and adult transcriptomic analysis suggests that the observed remodeling represents persistent molecular adaptations rather than acute pharmacological effects.

These observations are consistent with previous reports showing that SSRIs promote gradual transcriptional remodeling [8] and that sertraline can modify RNA processing across biological systems [9]. Together, our results support the idea that alternative splicing constitutes an additional post-transcriptional layer through which neuronal adaptation to sertraline may occur.

The higher prevalence of splicing events at increased sertraline concentrations further supports dose-dependent remodeling of RNA processing, suggesting that alternative splicing contributes to neuronal adaptation under different sertraline exposure conditions.

In this framework, the intersection analysis revealed that the sets of alternatively spliced genes differed among experimental conditions, with each dose×time combination exhibiting a distinct repertoire (Fig. 2). This suggests that sertraline induces condition-specific splicing responses shaped by the exposure regimen.

This pattern aligns with previous studies showing that sertraline induces persistent, gene-specific alternative splicing changes in the *Drosophila* nervous system, affecting transcripts associated with axonal stability, neuronal excitability, and cytoskeletal organization [5]. Similarly, antidepressants modulate alternative splicing in a dose- and time-dependent manner across biological systems, generating distinct isoform repertoires with prolonged exposure [10, 11].

Taken together, these findings suggest that the exclusive sets of alternatively spliced genes reflect distinct cellular adjustment states defined by dose and exposure duration. Therefore, alternative splicing may represent a fine-tuning mechanism that links different sertraline exposure conditions to neuronal functional state.

Our splicing analyses revealed that sertraline induces distinct modifications in mRNA architecture, including exon skipping, mutually exclusive exons, and alternative splice-site selection, that vary according to dose and exposure duration. Each experimental condition generated a distinct repertoire of structurally remodeled transcripts.

This pattern is consistent with the slow-adaptation model of SSRI action, in which therapeutic effects arise through gradual, plasticity-dependent transcriptional and post-transcriptional adjustments [12]. Additionally, sertraline has been shown to reshape mRNA structure in non-neuronal eukaryotes, particularly through retention intron and differential splice-site use, supporting the idea that RNA-processing reconfiguration is a structured cellular response [9]. However, the molecular mechanisms responsible for the splicing remodeling observed after sertraline exposure remain to be determined.

Critically, in *Drosophila*, larval exposure to sertraline induces persistent alternative splicing changes in adult neural tissue, affecting transcripts linked to cytoskeletal organization and synaptic dynamics [5]. Accordingly, the exon-skipping events we observed in PMCA, GPRK1, TROL, and SLO may represent context-specific transcriptomic adjustments associated with differential sertraline exposure.

The alternative splicing modifications detected across conditions may constitute fine regulatory mechanisms that contribute to neural functional adaptation under differential sertraline exposure.

It should also be considered that some of the truncated transcript isoforms may contain premature termination codons and therefore could be susceptible to nonsense-mediated mRNA decay (NMD). Consequently, not all reconstructed isoforms are expected to produce stable protein products, and their biological impact may also involve post-transcriptional regulation at the RNA level.

Because sertraline exposure occurred during larval stages while RNA sequencing was performed on adult heads, the transcriptomic states described here likely reflect persistent regulatory adjustments maintained across developmental stages. These findings indicate that early pharmacological exposure can reshape RNA processing programs that remain detectable in the mature nervous system.

Given that alternative splicing can modify not only transcript abundance but also protein architecture, we next examined whether these events may influence the functional domains of the affected proteins. This analysis focused on genes involved in calcium handling and synaptic organization, such as PMCA, whose domain composition directly determines their functional properties in neural tissue.

PMCA is a high-affinity P-type ATPase responsible for the precise extrusion of Ca²⁺ within neuronal microdomains, particularly in synaptic terminals and dendritic compartments where Ca²⁺ dynamics regulate plasticity, firing properties, and excitatory homeostasis [13]. Its C-terminal regulatory region, containing calmodulin-binding sites and other regulatory modules, tunes pumping kinetics in response to neuronal activity [14].

In our data, the 24 h isoform (T50mg) retains this C-terminal regulatory region, along with intrinsically disordered segments and compositionally biased regions, suggesting a protein conformation capable of dynamic modulation. In contrast, the 48 h isoform shows a complete loss of these regulatory elements, leaving the protein reduced to its catalytic core. Although Ca²⁺ extrusion capacity would be expected to be preserved, the protein’s ability to modulate activity in response to intracellular signaling cues may be constrained [15].

Previous studies have shown that alternative splicing at the C-site of PMCA determines the speed and amplitude of cytosolic Ca²⁺ clearance, directly influencing Ca²⁺-dependent forms of synaptic plasticity and the stabilization of firing patterns [16]. Moreover, the loss of regulatory regions increases PMCA vulnerability to oxidative stress and sustained functional impairment, as described in aging and neurodegenerative conditions [13, 17].

Within the context of sertraline exposure, these findings align with evidence demonstrating that SSRIs can disrupt Ca²⁺ homeostasis, promoting prolonged intracellular Ca²⁺ elevations, thereby increasing the demand for precise Ca²⁺ regulation by PMCA [1]. Under such physiological pressure, the shift toward a structurally simpler isoform may represent an alternative regulatory configuration whose functional consequences remain to be determined.

Collectively, these findings suggest that the structural modification of PMCA represents a context-dependent regulatory adjustment rather than a generalized loss of function. The 48 h isoform may therefore represent an alternative structural configuration within Ca²⁺ signaling pathways under sertraline exposure, although its effects on synaptic activity, excitability control, and long-term plasticity remain to be determined.

Gprk1 is a G-protein-coupled receptor kinase that regulates GPCR signaling through conserved catalytic and regulatory domains and is further modulated by the Ca²⁺ sensor recoverin, linking receptor signaling to synaptic adaptation [18, 19]. The 24 h (T200mg) isoform retains this domain organization. In contrast, the 48 h isoform is predicted to lack the RGS-like domain, kinase core, AGC regulatory region, and PH domain, suggesting altered receptor phosphorylation, membrane localization, and Ca²⁺-dependent regulatory feedback [19, 20].

These structural differences suggest altered regulatory properties. The predicted domain composition of the 48 h isoform may influence receptor signaling dynamics, consistent with the idea that alternative splicing can modify synaptic responsiveness in activity-dependent contexts [21]. Previous studies indicate that modulation of GRK activity alters synaptic plasticity and excitatory equilibrium in circuits exposed to sustained neuromodulatory signals, including antidepressants [22].

Thus, the predicted loss of regulatory domains in Gprk1 at 48 h may represent a transcriptional adjustment associated with prolonged sertraline exposure, potentially affecting GPCR signaling sensitivity, rather than a nonspecific or deleterious effect.

Trol encodes Perlecan, an extracellular matrix proteoglycan essential for synaptic organization, stability, and activity-dependent remodeling in Drosophila [23–26]. Recent studies have shown that disruption of Perlecan alters the extracellular microenvironments, directly affecting synaptic efficacy and the stability of neuronal connections [27].

The higher dose retains the structurally complete isoform, while the lower dose is associated with a truncated variant predicted to have reduced interactions with the extracellular matrix. The appearance of the truncated isoform at 50 mg may not reflect damage but rather a differential structural adjustment, consistent with models where the neural extracellular matrix acts as a plastic reservoir that expands or contracts depending on the intensity and duration of modulatory signals [24, 25].

Accordingly, retention of the full isoform at 200 mg may preserve a more complete extracellular interaction network, whereas the truncated isoform at 50 mg may represent an alternative structural configuration, suggesting that alternative splicing may contribute to differences in the structural organization of extracellular matrix components across exposure conditions.

The Slo gene encodes the BK channel, a Ca²⁺- and voltage-activated potassium channel whose C-terminal region contains regulatory elements, including the RCK domains and the “Calcium bowl”, that determine calcium sensitivity and neuronal excitability [28].

In our study, the isoform observed under the 200 mg (D–48 h) condition retains the complete RCK domains and the “Calcium bowl” region, consistent with preservation of calcium-sensitive regulation. In contrast, the isoform associated with 50 mg maintains the functional pore and the S7–S9 segments, but lacks the “Calcium bowl” and part of the distal RCK domain. Although potassium conductance is predicted to be preserved, this structural change may reduce Ca²⁺ sensitivity and the dynamic activation range, limiting the channel’s ability to couple intracellular signaling to excitability adjustments [29]. These observations are consistent with previous studies showing that alternative splicing is a major determinant of BK channel functional diversity and synaptic regulation [30]. Accordingly, retention of the complete isoform at 200 mg may preserve the calcium-sensitive regulatory architecture, whereas the truncated isoform at 50 mg may represent a distinct regulatory configuration associated with the loss of key calcium-responsive regions [31].

These findings suggest that alternative splicing may remodel the calcium-dependent regulatory architecture of Slo rather than causing a generalized loss of function. Although the physiological consequences remain to be established, future studies should determine how these isoforms contribute to neuronal activity and long-term synaptic adaptation.

Overall, the results show that sertraline induces a dose- and time-dependent reorganization of neuronal alternative splicing programs, with coherent effects on the architecture of isoforms involved in functional regulation. Because the analysis was performed on transcriptomic data from whole adult heads, the proposed functional implications are inferred from transcript organization and protein domain architecture. Nevertheless, the persistence of these splicing changes after larval exposure suggests long-lasting remodeling of RNA-processing programs in the mature nervous system.

Interestingly, several of the alternatively spliced genes identified in this study are involved in calcium handling and calcium-dependent neuronal regulation, including PMCA and Slo. Given that sertraline has been reported to influence intracellular Ca²⁺ homeostasis [1], and that calcium-dependent signaling regulates alternative splicing through Ca²⁺-responsive signaling pathways and RNA splicing regulatory factors [32, 33], these observations raise the possibility that the splicing remodeling observed here is linked to broader calcium-dependent adaptive responses.

Because sertraline increases extracellular serotonin through inhibition of the serotonin transporter, activation of serotonin receptors may initiate intracellular signaling. In *Drosophila*, serotonin receptors activate distinct intracellular pathways, and the 5-HT2B receptor has been implicated in serotonergic regulation of neuronal activity [34]. Given that 5-HT2 receptors are generally coupled to Gq-dependent signaling, this pathway could potentially engage Ca²⁺-dependent signaling mechanisms, thereby providing an upstream route through which serotonergic signaling could influence calcium-dependent regulation of alternative splicing. Together, these observations suggest a plausible mechanistic framework linking serotonergic signaling, calcium homeostasis, and alternative splicing remodeling following sertraline exposure.

Although this proposed mechanistic framework is biologically plausible, the molecular intermediates connecting serotonergic signaling, calcium homeostasis, and alternative splicing remain to be elucidated. Future studies should determine how these pathways interact to shape neuronal adaptation following sertraline exposure.

## Conclusion

Global analyses showed that alternative splicing events are not distributed uniformly across conditions, but instead form specific repertoires defined by the interaction between dose and exposure time. These differences were reflected not only in the number of genes affected, but also in the nature of the events, the direction of the changes (gain or loss of exons), and the selectivity of the genes involved, indicating that the response does not correspond to a generalized program, but rather to a fine-tuned adjustment dependent on the cellular state at the time of exposure. In this experimental design, sertraline exposure occurred during larval development whereas transcriptomic analyses were performed in adult neural tissue, indicating that the observed splicing programs reflect molecular adjustments that persist across developmental stages.

In the time-based comparisons, the observed pattern shows that full isoforms appear predominantly at 24 h, whereas predicted truncated isoforms become evident after 48 h of exposure, across the conditions examined. This behavior is observed in PMCA and Gprk1, where prolonged exposure leads to shortened variants predicted to lose the regulatory regions that allow their activity to be adjusted to intracellular signaling.

On the other hand, in the dose-based comparisons performed at both 24 h and 48 h, the pattern is consistent: In the representative transcripts analyzed, the high dose (200 mg) maintained the full isoforms, whereas the low dose (50 mg) was associated with truncated variants. However, the interpretation differs according to the duration of exposure. At 24 h, the full isoform of Trol may be consistent with the preservation of structural features associated with synaptic scaffolding, whereas at 48 h, as seen in Slo, the retention of the full isoform at 200 mg preserves protein regions predicted to participate in excitability control, in contrast to the truncated variant associated with 50 mg, which lacks some of these predicted structural elements.

Taken together, these findings indicate that sertraline does not act solely as a modulator of serotonin reuptake, but is also associated with gradual remodeling of transcript isoforms linked to proteins involved in Ca²⁺ homeostasis, synaptic matrix organization, and excitability control. Instead, the results suggest that alternative splicing may represent a condition-dependent molecular response associated with dose- and time-dependent exposure to sertraline. Because exposure occurred during larval development while RNA sequencing was performed on adult heads, these results suggest that early pharmacological exposure can induce persistent remodeling of RNA-processing programs that remain detectable in the mature nervous system.

## Supplementary Information

Below is the link to the electronic supplementary material.

Supplementary Material 1

## Data Availability

Data are available on Figshare platform, under the link https://figshare.com/s/0d08a1564167ecfb7062.
