# Supplementary material for: Larval exposure to sertraline induces dose- and time-dependent remodeling of neuronal alternative splicing in adult Drosophila melanogaster
Source: Mol Biol Rep. 2026 Jul 30;53(1):1306. doi: 10.1007/s11033-026-12452-z (PMC13423992; doi:10.1007/s11033-026-12452-z)
Supplement: Supplementary file 1 — Supplementary Material 1 [file 11033_2026_12452_MOESM1_ESM.docx]

**Supplementary Figure 1. Sashimi plots of exclusive genes in T-50mg (24→48h).** Representation of the five most significant alternative splicing events (FDR ≤ 0.05) identified in the T-50mg (24→48h) comparison. The selected genes are Btk29A (A3SS), mdy (A5SS), CG17600 (RI), Hibadh (CG15093) (RI), and CG14253 (A5SS). The plots illustrate exon junctions
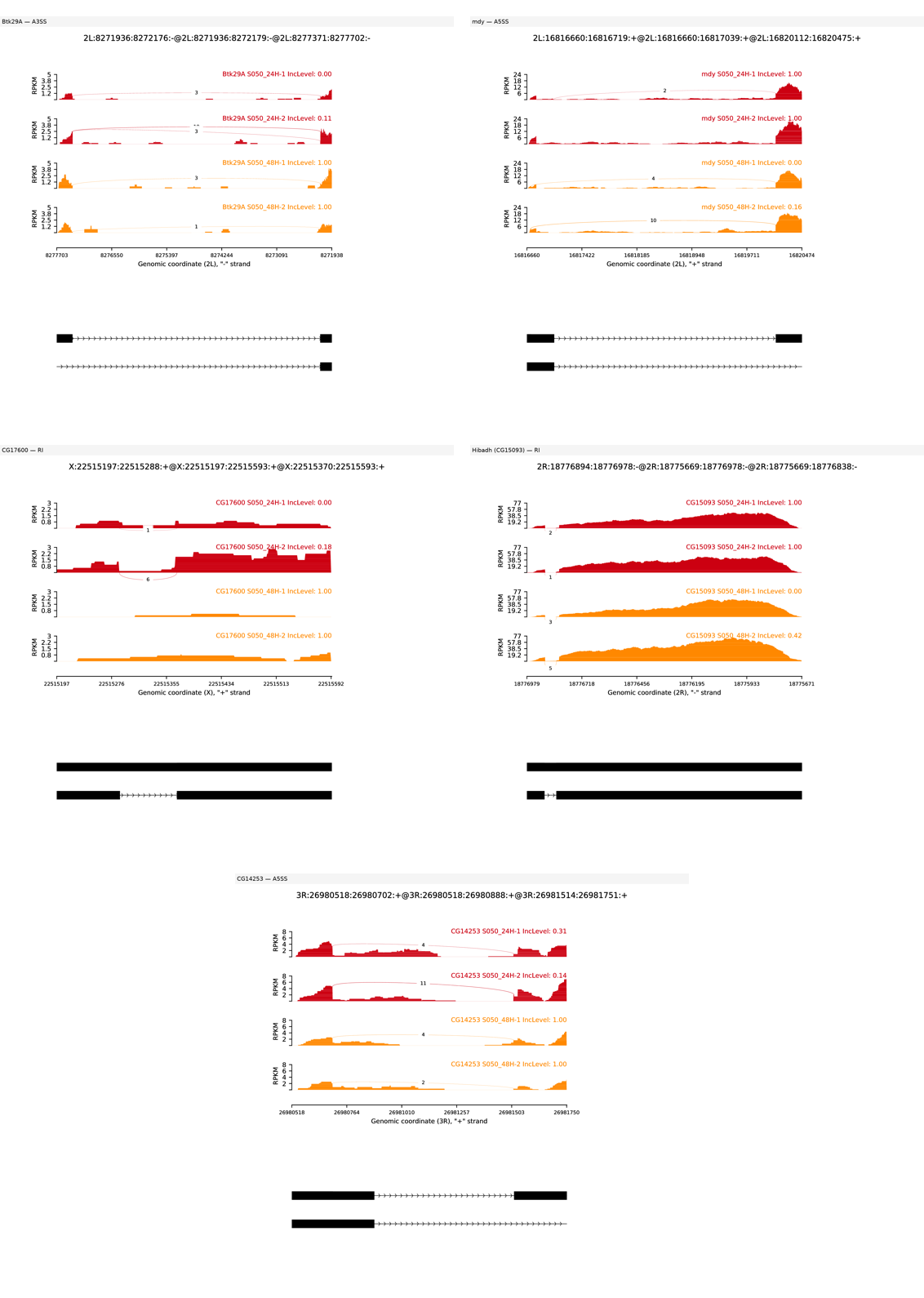
and read counts supporting each event.


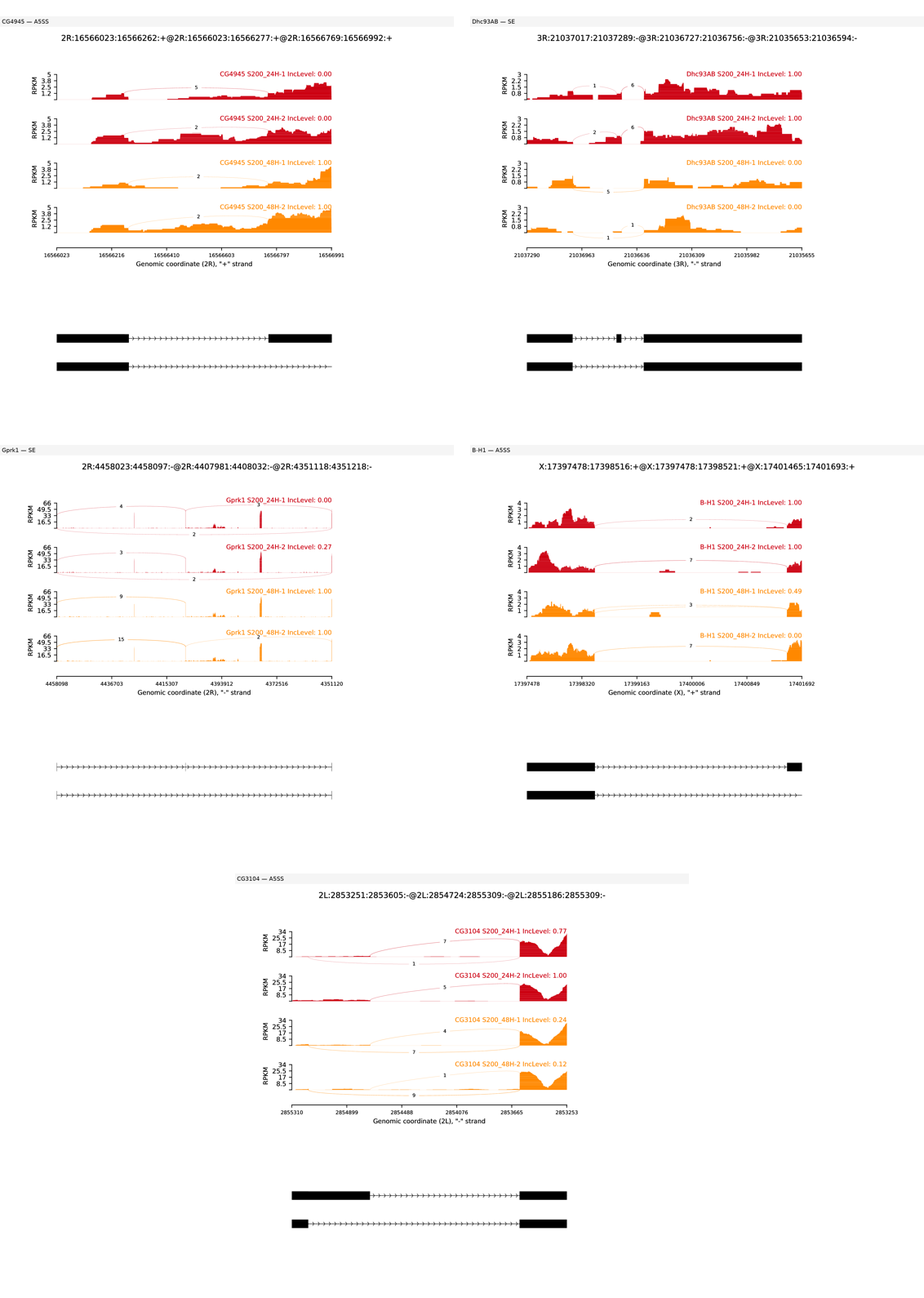

**Supplementary Figure 2. Sashimi plots of exclusive genes in T-200mg (24→48h).** Depiction of the five genes with exclusive alternative splicing events in the T-200mg (24→48h) comparison. These include CG4945 (A5SS), Dhc93AB (SE), Gprk1 (SE), B-H1 (A5SS), and CG3104 (A5SS). Each plot highlights the detected splicing variants and the magnitude of changes between conditions.


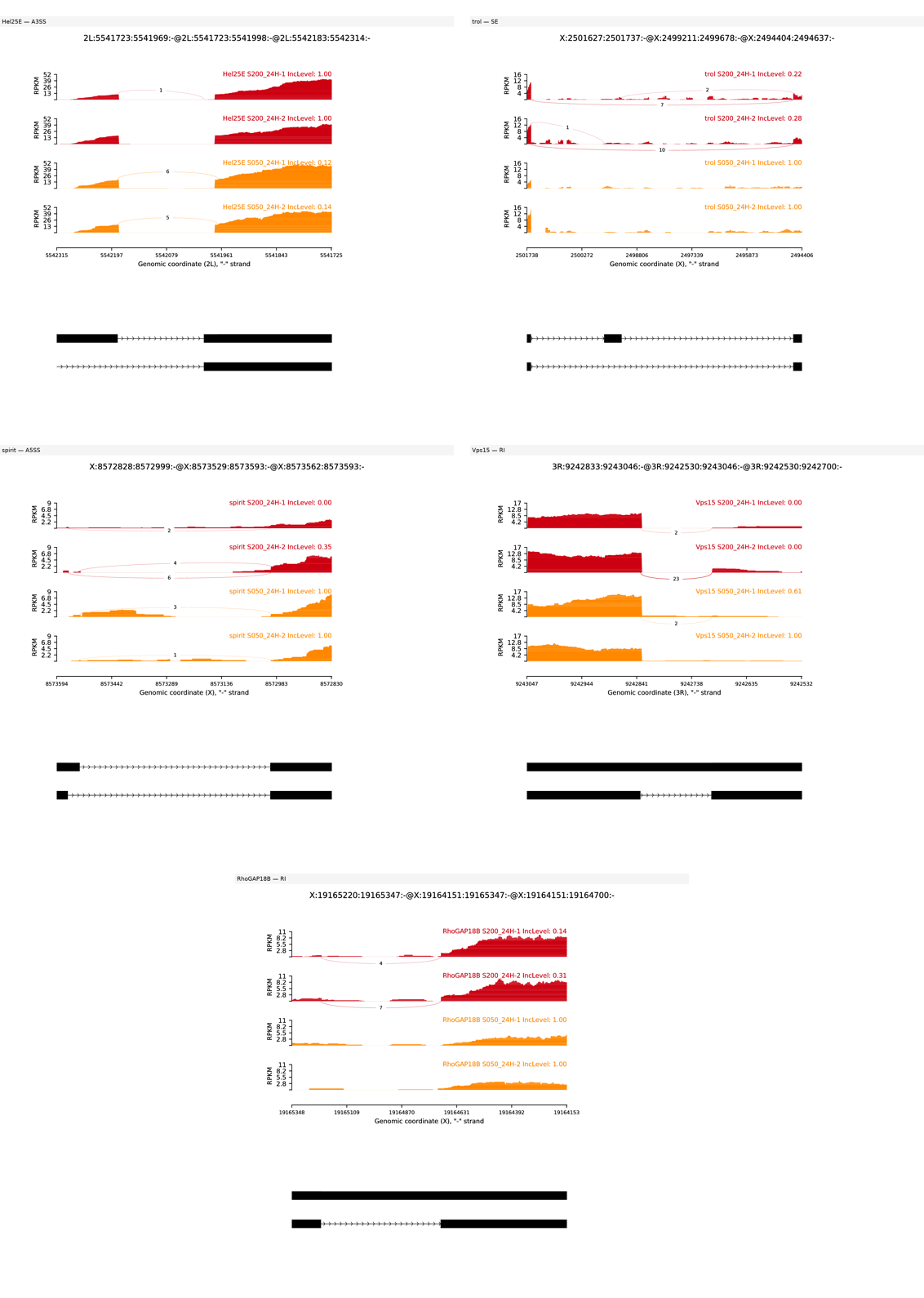


**Supplementary Figure 3. Sashimi plots of exclusive genes in D-24h (200 vs 50 mg).** The five selected genes for this condition were Hel25E (A3SS), trol (SE), spirit (A5SS), Vps15 (RI), and RhoGAP18B (RI). Each sashimi plot displays the alternative junctions and the differential inclusion proportion between experimental groups.


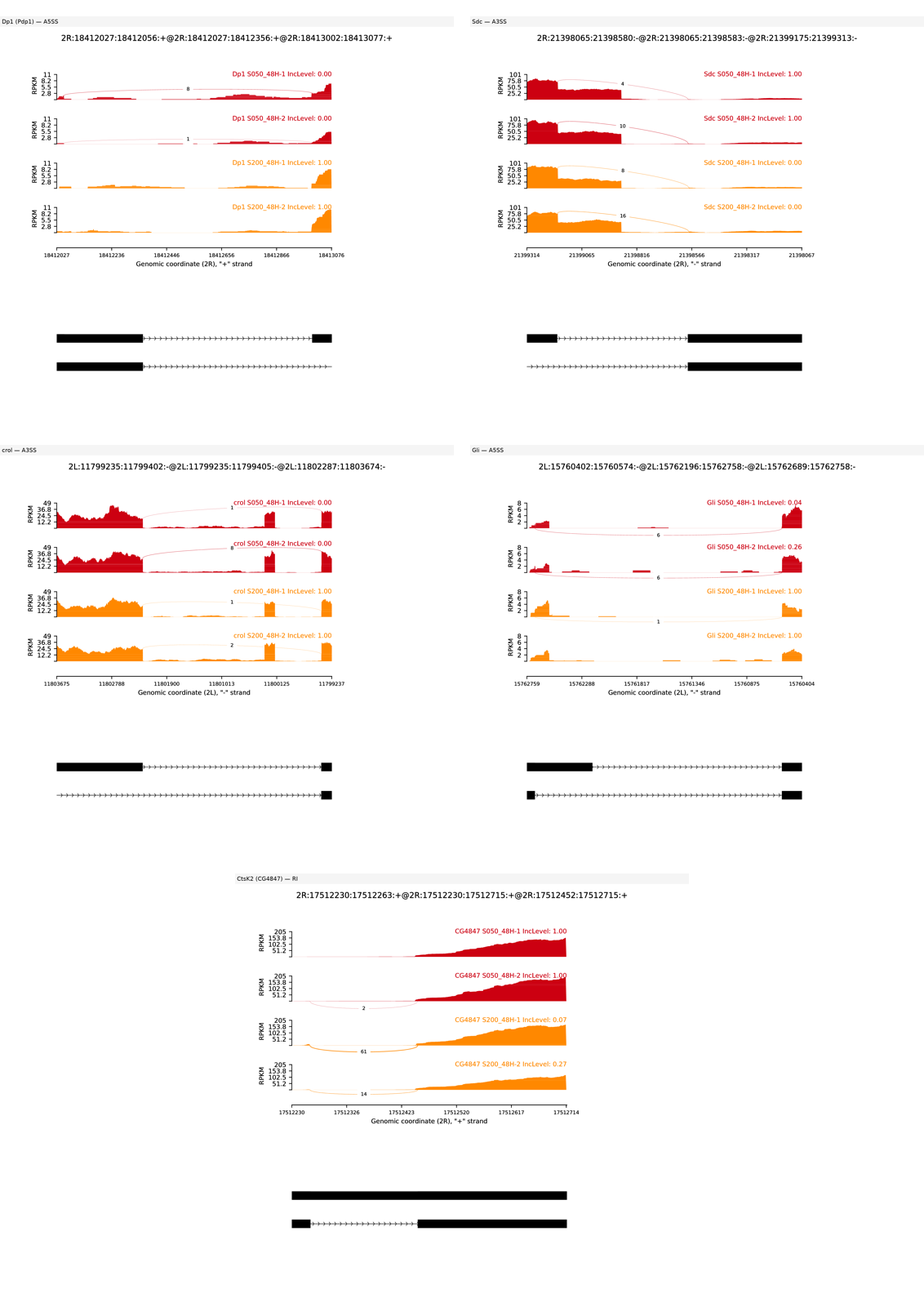


**Supplementary Figure 4. Sashimi plots of exclusive genes in D-48h (200 vs 50 mg).** The five genes with exclusive alternative splicing events in D-48h (200→50 mg) are shown: Dp1 (Pdp1) (A5SS), Sdc (A3SS), crol (A3SS), Gli (A5SS), and CtsK2 (CG4847) (RI). The plots depict differential splicing patterns together with read counts at splice junctions.
